# Dynamics of Telomerase-Based PD-L1 Circulating Tumor Cells as a Longitudinal Biomarker for Treatment Response Prediction in Patients with Non-Small Cell Lung Cancer

**DOI:** 10.3390/ijms26199583

**Published:** 2025-10-01

**Authors:** Issei Sumiyoshi, Shinsaku Togo, Takahiro Okabe, Kanae Abe, Junko Watanabe, Yusuke Ochi, Kazuaki Hoshi, Shoko Saiwaki, Shuko Nojiri, Yuichi Fujimoto, Yukiko Namba, Yoko Tabe, Yasuo Urata, Kazuhisa Takahashi

**Affiliations:** 1 Department of Respiratory Medicine, Juntendo University Graduate School of Medicine, 2-1-1 Hongo, Bunkyo-ku, Tokyo 113-8421, Japan; i-sumi@juntendo.ac.jp (I.S.); ju-watanabe@juntendo.ac.jp (J.W.); y-ochi@juntendo.ac.jp (Y.O.); s-saiwaki@juntendo.ac.jp (S.S.); yfujimo@juntendo.ac.jp (Y.F.); yknanba@juntendo.ac.jp (Y.N.); kztakaha@juntendo.ac.jp (K.T.); 2 Department of Minimally Invasive Next-Generation Cancer Diagnosis by TelomeScan, Juntendo University, 2-1-1 Hongo, Bunkyo-ku, Tokyo 113-8421, Japan; tabe@juntendo.ac.jp; 3 Leading Center for the Development and Research of Cancer Medicine, Juntendo University, 2-1-1 Hongo, Bunkyo-ku, Tokyo 113-8421, Japan; tokabe.scxm21@gmail.com (T.O.); k.hoshi.oe@juntendo.ac.jp (K.H.); 4 Oncolys BioPharma, Inc., 4-1-28 Toranomon, Minato-ku, Tokyo 105-0001, Japan; abe@oncolys.com (K.A.); urata@oncolys.com (Y.U.); 5 Medical Technology Innovation Center, Juntendo University, 2-1-1 Hongo, Bunkyo-ku, Tokyo 113-8421, Japan; s-nojiri@juntendo.ac.jp; 6 Department of Clinical Laboratory Medicine, Juntendo University Graduate School of Medicine, 2-1-1 Hongo, Bunkyo-ku, Tokyo 113-8421, Japan

**Keywords:** circulating tumor cell, epithelial–mesenchymal transition, non-small cell lung cancer, PD-L1, TelomeScan

## Abstract

Noninvasive liquid biopsy for monitoring circulating tumor cells offers valuable insights for predicting therapeutic responses. We developed TelomeScan^®^ (OBP-401), based on the detection of telomerase activity as a universal cancer cell marker and an indicator of the presence of viable circulating tumor cells (CTCs) for patients with advanced non-small cell lung cancer (NSCLC). This system evaluated CTC subtypes characterized by programmed death ligand 1 (PD-L1), an immune checkpoint molecule, and vimentin, an epithelial–mesenchymal transition (EMT) marker, using a multi-fluorescent color microscope reader. The prognostic value and therapeutic responses were predicted by dynamically monitoring CTC counts in 79 patients with advanced NSCLC. The sensitivity and specificity values of TelomeScan^®^ for PD-L1^(+)^ cells (≥1 cell) were 75% and 100%, respectively, indicating high diagnostic accuracy. PD-L1^(+)^ and EMT^(+)^ in CTCs were detected in 75% and 12% of patients, respectively. Detection of PD-L1^(+)^CTCs and PD-L1^(+)^EMT^(+)^ CTCs before treatment was associated with poor prognosis (*p* < 0.05). Monitoring of reducing and increasing PD-L1^(+)^ CTC counts in two sequential samples (baseline, cycle 2 treatment) correlated significantly with partial response (*p* = 0.032) and progressive disease (*p* = 0.023), respectively. Monitoring PD-L1^(+)^CTCs by TelomeScan^®^ will aid in anticipating responses or resistance to frontline treatments, optimizing precision medicine choices in patients with NSCLC.

## 1. Introduction

Detecting the presence of circulating tumor cells (CTCs) and determining their characteristics may help monitor the efficacy of systemic anticancer therapy and allow the early diagnosis of resistance to treatment, leading to the selection of more optimal therapeutic regimens for precision medicine [1,2,3]. Given their ability to improve clinical outcomes in patients with non-small cell lung cancer (NSCLC) while maintaining acceptable safety standards, immune checkpoint inhibitors (ICI) have been approved for use as first-line therapy for NSCLC [4,5].

ICIs can restore cancer immunity by targeting immune checkpoint proteins, such as programmed cell death-1 (PD-1), programmed death ligand 1 (PD-L1), and cytotoxic T-lymphocyte-associated protein 4 (CTLA-4). Both proteins are targeted during the first-line treatment of advanced lung cancer with ICI monotherapy or ICI combined with chemotherapy [6,7]. The PD-L1 expressed by CTC may serve as an indirect cancer-specific marker and may promote survival by allowing immune escape of cancer cells in the bloodstream. For NSCLC, %PD-L1 expression defined by the %tumor proportion score (TPS) is an indirect predictive marker for response to ICI treatment [8]; however, PD-L1 expression in CTCs remains controversial owing to the heterogeneity of CTCs. Furthermore, PD-L1 is associated with epithelial–mesenchymal transition (EMT), which promotes hematogenous cancer cell dissemination, metastatic relapse, resistance to treatment, and malignancy [9,10,11,12]. The longitudinally analysis of circulating cell surface vimentin-positive CTCs in the first two sequential samples (baseline, cycle 4) following treatment has been reported to be a significant predictor of earlier relapse in patients with neuroblastoma [13]. The gene promoter region of PD-L1 contains a binding site for the ZEB1 transcription factor, which induces EMT, suggesting that PD-L1 expression coordinates with EMT, resulting in tumor progression [14]. Therefore, EMT-induced immune escape promotes tumor progression with higher PD-L1 expression [15]. These marker subtypes expressed on CTCs provide valuable clinical information to overcome challenges in tumor biopsy spatiotemporal heterogeneity.

The CellSearch System^®^ (Veridex LLC, Raritan, NJ, USA) is widely available for CTC detection using immunomagnetic cell enrichment [2]. However, the detection method relies on capturing epithelial surface antigens on CTCs, including epithelial cell adhesion molecules, which are relatively unlikely to be detected on EMT^(+)^ CTCs. Telomerase activity is a representative cancer-universal marker (high in >90% of malignancies) and reflects the presence of live cancer cells. Thus, telomerase measurement in multiple cancer types has been reported as a tumor marker with demonstrated diagnostic and prognostic utility [16,17,18,19,20]. High telomerase activity assayed from live-captured CTCs using the parallel enumeration by CellSearch System^®^ was prognostic of worse overall survival (OS) in patients with metastatic castration-resistant prostate cancer. These results illustrate the potential of CTC-derived telomerase activity as a predictive marker for cancer prognosis and treatment response [21]. The TelomeScan^®^ (OBP-401) assay is a distinct, highly sensitive, and viable CTC detection system that relies on telomerase activity, a critical step in carcinogenesis that correlates with human telomerase reverse transcriptase (hTERT) expression [9,10,11,12]. The TelomeScan^®^ assay is based on a selective telomerase replication adenovirus that uses hTERT promoter-mediated viral replication to visualize viable CTCs that are green fluorescent protein (GFP)-positive and CD45^(−)^ among numerous normal cells. The TelomeScan^®^ is a useful biomarker for diagnosis and prognosis and has been used to predict therapeutic efficacy in various solid cancers [12,16,17,18,19,20]. Furthermore, as the telomerase-dependent adenovirus used in TelomeScan^®^ selectively infects PD-L1^(+)^ CTCs, we highlight the potential of this approach not only as a predictive biomarker but also as a virotherapy directly targeting PD-L1^(+)^ tumor cells in the circulation [22,23]. However, the usefulness of CTC assays in clinical settings for monitoring lung cancer therapy is limited as clinical data has been inconclusive. Herein, we aimed to identify PD-L1^(+)^ EMT^(+)^ CTCs in patients with NSCLC using the TelomeScan^®^ assay and to investigate whether the PD-L1^(+)^ EMT^(+)^ CTC count can be used as an early predictor of responses to frontline treatment and prognosis.

## 2. Results

### 2.1. Patient Characteristics

**Figure 1:**
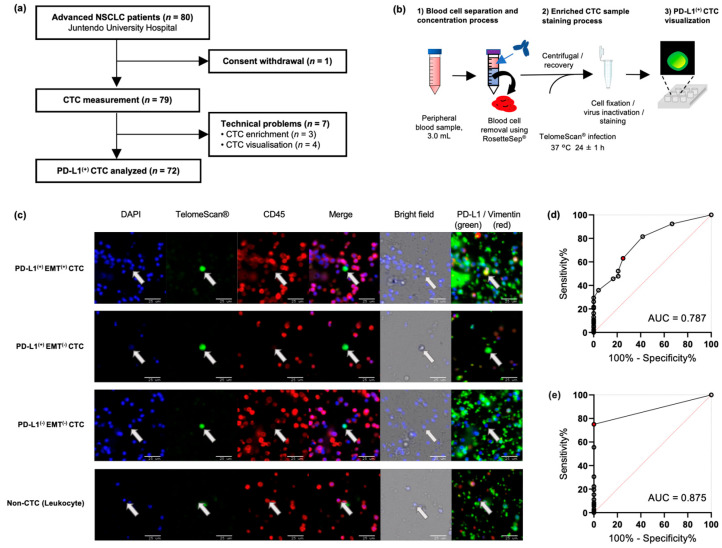
Highly sensitive circulating tumor cell subtype detection by TelomeScan^®^. (**a**) Study flow chart of advanced NSCLC patients (*n* = 80) in this cohort study. (**b**) Schematic consisting of three steps: (**b1**) concentration of circulating tumor cell (CTC)-enriched blood; (**b2**) incubation with TelomeScan^®^ (OBP-401) and staining of phenotypic markers by immunocytochemistry; (**b3**) analysis of the CTC images and counts using a multi-fluorescent color microscope reader. (**c**) Representative TelomeScan-based multicolor images of CTC subtypes: PD-L1^(+)^ EMT^(+)^, PD-L1^(+)^ EMT^(−)^, PD-L1^(−)^ EMT^(−)^, and a leukocyte (non-CTC). EMT positivity was defined by vimentin expression. (**d**) Receiver operating characteristic (ROC) curve was used to determine the cut-off value for CTCs as 3 mL blood ≥ 3 CTCs diagnosis of non-small-cell lung cancer (NSCLC). The red dotted line indicates the point with the maximum Youden Index. The sensitivity and specificity of CTC analysis in the diagnosis of NSCLC were 63.0% and 75.0%, respectively. (**e**) ROC curve was used to determine the cut-off value for PD-L1^(+)^ CTCs. The red dotted line indicates the point with the maximum Youden Index. Sensitivity and specificity of PD-L1^(+)^ CTC analysis in the diagnosis of NSCLC achieved 75.0% and 100%, respectively. EMT, epithelial–mesenchymal transition; PD-L1, programmed death-ligand 1; AUC, area under the curve.

Of the 80 patients with NSCLC who provided their written informed consent, one patient withdrew consent and was excluded from the study (Figure 1a). Thus, peripheral venous blood samples were obtained from 79 patients with pathologically proven NSCLC (Supplemental Data S1). All patients with advanced or relapsed NSCLC received ICI with chemotherapy or ICI or chemotherapy alone. Eleven patients with NSCLC harboring driver mutations, who had been treated with tyrosine kinase inhibitors, were enrolled after disease relapse.

### 2.2. Highly Sensitive Detection of CTC Subtypes by TelomeScan^®^

Representative fluorescent images illustrating PD-L1^(+)^ EMT^(+)^, PD-L1^(+)^ EMT^(−)^, PD-L1^(−)^ EMT^(−)^, and leukocyte (non-CTC) cells are shown in Figure 1c. Viable CTCs present as single cells with no previously reported circulating tumor micro-emboli [24]. PD-L1^(+)^ CTCs were not detected in any of the healthy controls (*n* = 13) or in patients with benign lung disease (*n* = 11), whereas they were found in 75% (*n* = 54) of patients with NSCLC (2.94 ± 4.73 cells/3 mL). The total CTC levels and PD-L1^(+)^ CTC levels of patients with NSCLC were significantly higher than those of healthy individuals (CTCs, *p* = 0.006; PD-L1^(+)^ CTCs, *p* < 0.001) and of patients with non-carcinomatous lung disease (CTCs, *p* = 0.009; PD-L1^(+)^ CTCs, *p* < 0.001), whereas EMT^(+)^ CTCs were not detected in control individuals (Supplemental Data S2). Receiver operating characteristic (ROC) curve analysis indicated that a threshold of ≥3 CTCs/3 mL blood provided 63.0% sensitivity and 75.0% specificity for the diagnosis of NSCLC (Figure 1d). In comparison, the detection of PD-L1^(+)^ CTCs demonstrated higher diagnostic accuracy, with a sensitivity of 75% and specificity of 100% (Figure 1e). Accordingly, subsequent analyses focused on the clinical utility of PD-L1^(+)^ CTCs.

### 2.3. CTC Detection and Association with Clinicopathological Subtypes

PD-L1^(+)^ CTCs were evaluated in 72 of the 79 patients prior to treatment. In the remaining seven patients, PD-L1^(+)^ CTC enumeration was not possible due to technical errors or coagulation of peripheral blood samples. Among the evaluable patients, 75% (n = 54) exhibited PD-L1^(+)^ CTCs, while 25% (*n* = 18) did not. Importantly, 91.7% (*n* = 12) of patients with EMT^(+)^ (=vimentin^(+)^) CTCs demonstrated double-positive staining for PD-L1, suggesting that CTCs undergoing EMT exhibited PD-L1 expression (Table 1). The presence of PD-L1^(+)^ EMT^(+)^ CTCs was significantly (*p* = 0.030) associated with heavy smoking. The %TPS of individual tumor biopsy samples and the corresponding %PD-L1^(+)^ CTC samples were not correlated (Supplemental Data S3).

### 2.4. Association of Baseline PD-L1^(+)^ CTC Measurements with Prognostic Outcome

**Figure 2:**
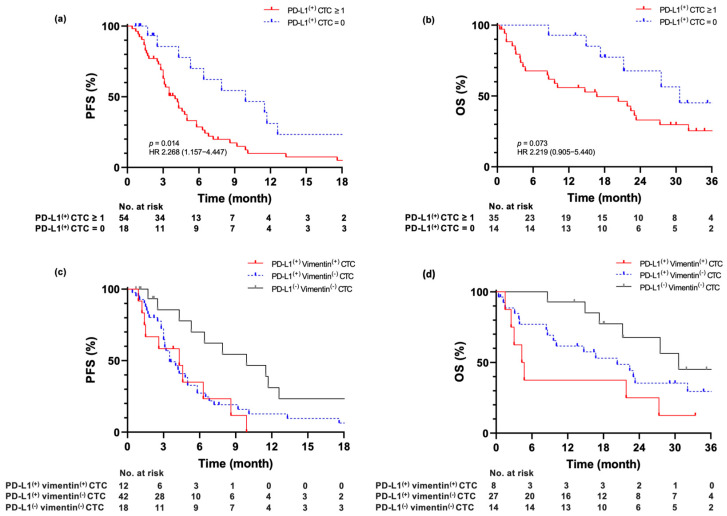
Association of circulating tumor cell measurement with progression-free survival and overall survival. (**a**,**b**) Comparison of patients with programmed death−ligand 1 (PD-L1)^(+)^ CTCs/3.0 mL of blood: 0 vs. ≥1 CTCs. (**c**,**d**) Further stratification of patients according to EMT status: PD-L1^(−)^ vimentin^(−)^ CTCs, PD-L1^(+)^ vimentin^(−)^ CTCs, or PD-L1^(+)^ vimentin^(+)^ CTCs/3.0 mL of blood. *p*-values were calculated using the log-rank test. The hazard ratio (HR) was calculated using the Cox proportional hazards model.

The median progression-free survival (PFS) in patients without PD-L1^(+)^ CTC, regardless of the total CTC count, was 9.9 months (95% CI = 4.0–15.8, *n* = 18), whereas in those with PD-L1^(+)^ CTC was 4.0 months (95% CI = 3.0–5.0, *n* = 54; *p* = 0.014). The median OS was not reached in patients without PD-L1^(+)^ CTCs compared with those with PD-L1^(+)^ CTCs (*p* = 0.073). (Figure 2a,b).

Furthermore, when patients were stratified according to EMT marker expression, three subgroups were identified: double negative [PD-L1^(−)^ EMT^(−)^], single positive [PD-L1^(+)^ EMT^(−)^], and double positive [PD-L1^(+)^ EMT^(+)^]. In this stepwise analysis, the median PFS of patients with PD-L1^(-)^ EMT^(-)^ CTCs was 9.9 months (95% CI = 4.0–15.8, *n* = 18), and that of those with PD-L1^(+)^ EMT^(+)^ CTCs was 4.3 months (95% CI = 1.7–6.9, *n* = 12; *p* = 0.006). The median OS of treatment-naïve patients with PD-L1^(-)^ EMT^(-)^ CTCs was 30.6 months (95% CI = NA, *n* = 18), and that of patients with PD-L1^(+)^ EMT^(+)^ CTCs was 4.3 months (95% CI = 1.9–6.7, *n* = 12; *p* = 0.015) (Figure 2c,d). The ratio of PD-L1^(+)^ CTCs for all CTCs (PD-L1^(+)^ CTCs/all CTCs ≥ 50%) was not associated with PFS in patients treated with regimens, such as those including ICIs (Supplemental Data S4).

### 2.5. PD-L1^(+)^ CTC Count Monitoring to Predict Therapeutic Response and Prognostic Outcome

**Figure 3:**
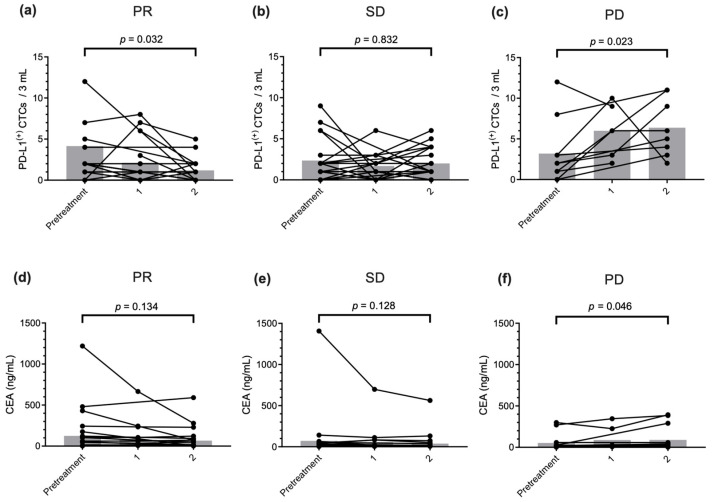
Prediction of treatment response by circulating tumor cell counts and serum CEA level monitoring. Changes in programmed death-ligand 1 (PD-L1)^(+)^ CTC counts before and after the two cycles of therapy. (**a**) PR; (**b**) SD; (**c**) PD. Changes in measured serum CEA levels before and after the two cycles of therapy. (**d**) PR; (**e**) SD; (**f**) PD. *p*-values were calculated using the Wilcoxon signed-rank test.

A significant decrease in the PD-L1^(+)^ CTC counts from 4.2 (range: 0.0–34.0) to 1.2 (range: 0.0–5.0) cells was observed in patients with partial response (PR) after two cycles of treatment (*p* = 0.032, Figure 3a); however, it did not change in patients with stable disease (SD) (*p* = 0.832, Figure 3b). At baseline (before treatment), significantly increased PD-L1^(+)^ CTC counts, from 3.2 (range: 0.0–12.0) to 6.2 (range: 2.0–11.0) cells, were observed in patients with PD after two treatment cycles (*p* = 0.023, Figure 3c). The change in serum carcinoembryonic antigen (CEA) levels in patients with PR and SD was not significant (PR: *p* = 0.134, SD: *p* = 0.128, Figure 3d,e). However, a significant increase in serum CEA levels was observed in patients with PD after two treatment cycles, which was similar to the change in the PD-L1^(+)^ CTC count (*p* = 0.046, Figure 3f). The PD-L1^(+)^ CTC counts were significantly elevated at all time points of PD compared with the pretreatment counts (*p* = 0.016, see Figure, Supplemental Data S5).

Increasing PD-L1^(+)^ CTC levels between pretreatment and two cycles post-treatment was associated with poor PFS (*p* = 0.040, Supplemental Data S6). The median PFS of patients with increasing PD-L1^(+)^ CTCs was 5.8 months (95% CI = 2.1–9.5, *n* = 19), whereas that of patients with decreasing PD-L1^(+)^ CTCs was 10.3 months (95% CI = 6.1–14.5, *n* = 29). These results suggest that monitoring PD-L1^(+)^ CTC could be an early and sensitive predictor of treatment response.

## 3. Discussion

In this study, we developed and adapted a highly sensitive/specific CTC detection system using TelomeScan^®^ (OBP-401) and evaluated CTC populations expressing PD-L1^(+)^ EMT^(+)^. The assay achieved high sensitivity and specificity in PD-L1^(+)^ CTC measurements, thus demonstrating its high diagnostic value in patients with NSCLC.

The baseline detection of total CTC count using the TelomeScan^®^ assay has been reported to be beneficial in the diagnosis, prognosis prediction, and evaluation of therapeutic efficacy in small-cell lung, breast, gastric, and gynecological cancers [16,17,20,25]. CTC detection systems using antigen-independent methods for NSCLC have also been proposed. Ficoll density gradient centrifugation was used to isolate detected CTCs and PD-L1^(+)^ CTCs in 94% and 86% of patients with advanced NSCLC at baseline, respectively [26]. Similarly, the ISET^®^ assay, which isolates tumor cells based on size, identified CTCs and PD-L1^(+)^ CTCs in 80% and 60.8% of advanced NSCLC patients, respectively [27]. Although the reported detection rates are high, the absence of specificity data limits the interpretability of these results, as Ficoll-based enrichment or the ISET^®^ assay may allow contamination with non-malignant mononuclear cells. The widely used epithelial antigen-dependent CTC detection system CellSearch^®^ was able to detect CTCs in 22.5–43.4% of patients and PD-L1^(+)^ CTCs in 9.4% of patients, suggesting a lower probability of detecting EMT^(+)^ CTC. However, the concordance of PD-L1 expression between tumor tissue and CTCs is low [28,29,30]. Furthermore, we observed no correlation between %PD-L1^(+)^ CTC and %TPS, which may be attributed to the heterogeneity of CTCs.

The clinical utility of PD-L1^(+)^ CTCs as biomarkers to identify patients suitable for PD-L1 blockade therapy has been reported [29,31,32,33]. More recent studies further demonstrated that PD-L1 expression on CTCs is associated with epithelial–mesenchymal transition and can predict therapeutic efficacy of immune checkpoint inhibitors [34,35]. The reduction in PD-L1^(+)^ CTC counts is associated with a beneficial response to PD-1/PD-L1 inhibitors, and gastrointestinal cancer patients with PD-L1^(+)^ CTCs have longer survival than those with PD-L1^(-)^ CTCs [36]. Prediction of resistance to PD-1/PD-L1 inhibitors is possible in patients with elevated PD-L1^(+)^ CTCs [26,30]. However, our results demonstrated that a high ratio of PD-L1^(+)^ CTCs (≥50%) is not associated with prolonged PFS in patients treated with regimens, including ICIs. Conversely, PD-L1^(+)^ CTCs have been associated with poor clinical outcomes in patients treated with PD-1 inhibitors [37], indicating that the diagnostic value of PD-L1^(+)^ CTC measurements with respect to predicting the response to PD-L1 blockade treatment remains controversial.

We found that baseline PD-L1^(+)^ CTC levels and elevated PD-L1^(+)^ CTC counts during frontline treatment with or without ICI may serve as valuable early and sensitive predictors of poor prognosis and disease recurrence. The combination of CTC count and serum tumor marker monitoring has been suggested as a useful tool to predict early recurrence in patients with advanced NSCLC [38,39]. However, this study found that monitoring the PD-L1^(+)^ CTC counts from baseline to two cycles post-treatment was more sensitive to predicting treatment responses than monitoring serum CEA levels alone. In this respect, TelomeScan^®^ (OBP-401), the telomerase activity-dependent CTC detection system, provides direct evidence of viable cancer cells and can contribute helpful information on the current cancer status of patients.

Vimentin expression is positively correlated with increased PD-L1 levels at NSCLC recurrence [40]. The smoking-mediated immune escape via PD-L1 pathways induced by a high tumor mutational burden has been associated with EMT and contributes to more malignant CTC phenotypes [41,42,43]. EMT^(+)^ CTCs were detected in 12 patients, and PD-L1 was co-expressed in 11 of these cases (91.7%). These cells were more frequently observed in heavy smokers and appeared to be associated with shorter PFS and OS. These results confirm those of previous studies showing that PD-L1^(+)^ EMT^(+)^ CTCs are associated with poor outcomes, including various smoking-related cancers [44,45,46].

This study had certain limitations. First, this study was performed at a single institution; however, the threshold value of the PD-L1^(+)^ CTC count as a prognostic factor was not independently validated at another institution. Second, the study population involved patients who underwent diverse treatment regimens, including chemotherapy and ICI combination therapies, or chemotherapy alone, and it excluded patients treated with molecular target tyrosine kinase inhibitors. Therefore, a further large prospective multi-institutional validation study is required to confirm our results and to determine whether PD-L1^(+)^ EMT^(+)^ CTC monitoring could help predict response or resistance to different types of therapies, including anti-PD-1/PD-L1 inhibitors and molecularly targeted tyrosine kinase inhibitors.

TelomeScan^®^-guided PD-L1^(+)^ CTC measurements are highly detectable in patients with NSCLC. Monitoring PD-L1^(+)^ CTC measurements can predict early sensitive treatment responses and has potential for therapeutic application to identify patients with the highest probability of achieving disease-free status.

## 4. Materials and Methods

### 4.1. Study Design

This cohort study adhered to the STROBE guidelines [47]. The study was conducted at the Juntendo University Hospital from April 2020 to December 2021 (Tokyo, Japan). All experiments were conducted in accordance with the Declaration of Helsinki. Patients who met the following criteria were recruited: (i) histologically- or cytologically confirmed NSCLC with clinical staging based on chest radiography, computed tomography (CT), brain magnetic resonance imaging (MRI) and positron emission tomography (PET) findings; (ii) evaluable or measurable disease; and (iii) with no active concomitant malignancy. Tumor response was classified using the response evaluation criteria for solid tumors (version 1.1) [48]. Tumor progression was routinely assessed using CT, brain MRI, or PET scans.

CTCs were detected in peripheral blood specimens (3 mL) within 14 days before commencing treatment (baseline) and monitored at each of the three time points, one, two, and four standard treatment cycles, until progressive disease (PD) was diagnosed. Blood samples were collected from healthy volunteers and from patients with non-carcinomatous lung diseases, such as lung fibrosis and non-tuberculosis mycobacterium.

### 4.2. TelomeScan^®^-Guided CTC Visualization

OBP-401 (TelomeScan^®^; Oncolys BioPharma Inc., Tokyo, Japan) infection induces a telomerase-specific, replication-selective, oncolytic adenovirus, producing GFP^(+)^ cells for viable CTC selection [49,50]. The CTC detection system using TelomeScan^®^ (OBP-401) is shown in Figure 1b. Peripheral blood samples were drawn into an ethylenediaminetetraacetic acid disodium (EDTA-2Na) tube (Cat No. VP-NA070KN60; Terumo, Tokyo, Japan) and stored at 25 °C until use. Testing procedures began within 12 h of collection. Briefly, 3 mL peripheral blood in the EDTA tube was transferred into a 15 mL tube and mixed with an on-chip T-buffer solution (Cat No. 2001014, On-chip Biotechnologies, Tokyo, Japan) consisting of 2% fetal calf serum (FCS), EDTA solution and human FcR blocking reagent (Cat No. 130-059-901; Miltenyi Biotec, Bergisch Gladbach, Germany). After 10 min of incubation at 25 °C, the RosetteSep™ CTC Enrichment Cocktail containing Anti-CD36 (Cat No. ST-15167; Veritas, Tokyo, Japan) was added to remove blood cells. The sample mixture was poured onto a density gradient SepMate™ (Cat No. ST-86415, Veritas) and centrifuged at 500× *g* and 25 °C for 15 min. The supernatant was collected in a 50 mL tube and subsequently diluted with 2% FCS/PBS. After platelets were eliminated by centrifugation (200× *g* for 10 min), the cell pellet was washed and suspended in Dulbecco’s modified Eagle medium containing 10% FCS. The enriched CTC fraction was incubated at 37 °C with TelomeScan^®^ (OBP-401) (10^9^ virus particles per sample) for 24 h. After infection, the medium was diluted with 2% FCS/T buffer and centrifuged at 500× *g* for 5 min. The supernatant was discarded, and the cell pellet was suspended in 4% paraformaldehyde (Cat No. 09154-85, Nacalai-Tesque, Kyoto, Japan) and 0.15% Triton X-100 (Cat No. 93343-100ML, Sigma, St. Louis, MO, USA) for 30 min at 25 °C, followed by virus inactivation, cell fixation, and immunostaining. After 10 min of incubation, the cell pellet was washed twice and resuspended in a 2% FCS/T-buffer containing the following conjugated antibodies: Alexa Fluor^®^ 647 anti-human CD45 antibody (1:100; Cat No. 304018; Biolegend, San Diego, CA, USA), PE anti-human PD-L1 antibody (1:400; Cat No. ab209962; Abcam, Cambridge, UK), and Alexa Fluor^®^ 750 anti-human/mouse/rat vimentin antibody (1:40; Cat No. IC2105S; R&D Systems, Minneapolis, MN, USA). The cells obtained were stained with DAPI (DOJINDO, Kumamoto, Japan) and seeded in a 96-well plate precoated with poly-L-lysine. OBP-401-infected GFP^(+)^ cells were counted, and false-positive cells were discriminated based on their anti-CD45 staining status: false-positive cells (GFP^+^/CD45^+^) were subtracted from the analysis. PD-L1 (defined as PD-L1^(+)^) and vimentin (defined as EMT^(+)^) levels in CTC were evaluated under a multifluorescence color microscope BZ-X810 (Keyence, Osaka, Japan). The wide-field images were acquired at 20× magnification to enable scanning of the extensive imaging area required to cover each well. Using higher magnifications would have markedly increased the acquisition time and data volume, making comprehensive scanning less feasible. Thus, 20× was selected as an appropriate balance between coverage and resolution. Importantly, the strong and distinct GFP signal produced by TelomeScan allowed reliable identification of CTCs at this magnification, ensuring accurate and reproducible enumeration and phenotypic characterization.

### 4.3. Statistical Analysis

Fisher’s exact test for categorical variables was used to assess the relationship among the number of CTCs, clinical characteristics, and treatment response. The Mann–Whitney U test or the Wilcoxon signed-rank test was used to compare two groups with asymmetrical sample distributions. The Kruskal–Wallis test and post hoc analysis (Dunn–Bonferroni test) were used to compare three groups with asymmetrical sample distributions. To evaluate the diagnostic performance of PD-L1^(+)^ CTC, we calculated the area under the ROC curves (AUC). PFS and OS were analyzed using the Kaplan–Meier method, log-rank test, and Cox proportional hazards regression. Bonferroni correction was used to compare groups. For each test, two-tailed *p* < 0.05 indicated statistical significance. All data were analyzed using SPSS v.29.0 (IBM Corp., Armonk, NY, USA).

## Figures and Tables

**Table 1:** Correlations between the circulating tumor cell (CTC) subtype and clinicopathological variables.

Patient Characteristics	PD-L1^(+)^ CTC *n* = 54		PD-L1^(−)^ CTC *n* = 18	*P*-Value
	Vimentin^(+)^ *n* = 12	Vimentin^(−)^ *n* = 42		
Age, median (range), years	69 (46–85)	70 (40–88)	70 (52–87)	0.755 ^a^
Sex				0.052 ^b^
Male	11	27	16	
Female	1	15	2	
Smoking history				0.030 ^b^
Heavy smoker (BI ≥ 400)	12 ^†^	28 ^†^	15	
Light/never smoked (BI < 400)	0 ^†^	14 ^†^	3	
Stage				0.028 ^b^
I, II, IIIA (Postoperative recurrence)	5	5 ^†^	8 ^†^	
IIIB	1	3	2	
IIIC	0	0	1	
IVA	3	16	5	
IVB	3	18 ^†^	2 ^†^	
Histology at diagnosis				0.121 ^b^
Adenocarcinoma	8	32	8	
Squamous cell carcinoma	2	5	7	
Others	2	5	3	
PD-L1 status				0.937 ^b^
TPS < 1%	5	14	8	
TPS 1–49%	2	9	3	
TPS ≥ 50%	4	17	7	
Unknown	1	2	0	
Line of therapy				0.690 ^b^
1	8	27	14	
2	2	9	1	
≥3	2	6	3	
Regimen				0.080 ^b^
ICI + chemotherapy	2	20	7	
ICI	3	9	8	
Chemotherapy	7	13	3	
Driver mutation				0.953 ^b^
EGFR	1	5	1	
ALK	0	0	0	
ROS-1	0	1	0	
BRAF	0	1	0	
Negative/unknown	11	35	17	
RECIST (2 courses)				0.716 ^b^
PR	6	12	6	
SD	2	16	7	
PD	3	8	2	
Sensor	1	6	3	

Abbreviations: CTC: circulating tumor cell, BI, Brinkman’s index; PD-L1, programmed cell death/programmed cell death-ligand-1; TPS, tumor proportion score; ICI, immune checkpoint inhibitor; EGFR, epidermal growth factor receptor; ALK, anaplastic lymphoma kinase; RECIST, response evaluation criteria in solid tumors; PR, partial response; SD, stable disease; PD, progressive disease. ^a^ *p*-values were calculated using the Kruskal–Wallis or chi-square tests. ^b^ *p*-values were calculated using Fisher’s exact test. ^†^ Adjusted standardized residuals > |1.96|.

## Data Availability

The datasets generated and/or analyzed in the present study are available from the corresponding author upon reasonable request.

## Supplementary Materials

The following supporting information can be downloaded at: https://www.mdpi.com/article/10.3390/ijms26199583/s1.
