# Supplementary material for: Dynamics of Telomerase-Based PD-L1 Circulating Tumor Cells as a Longitudinal Biomarker for Treatment Response Prediction in Patients with Non-Small Cell Lung Cancer
Source: Int J Mol Sci. 2025 Oct 1;26(19):9583. doi: 10.3390/ijms26199583 (PMC12524628; doi:10.3390/ijms26199583)
Supplement: Supplementary file 1 [file ijms-26-09583-s001.zip › ijms-3815377-supplementary.pptx]

## Slide 1
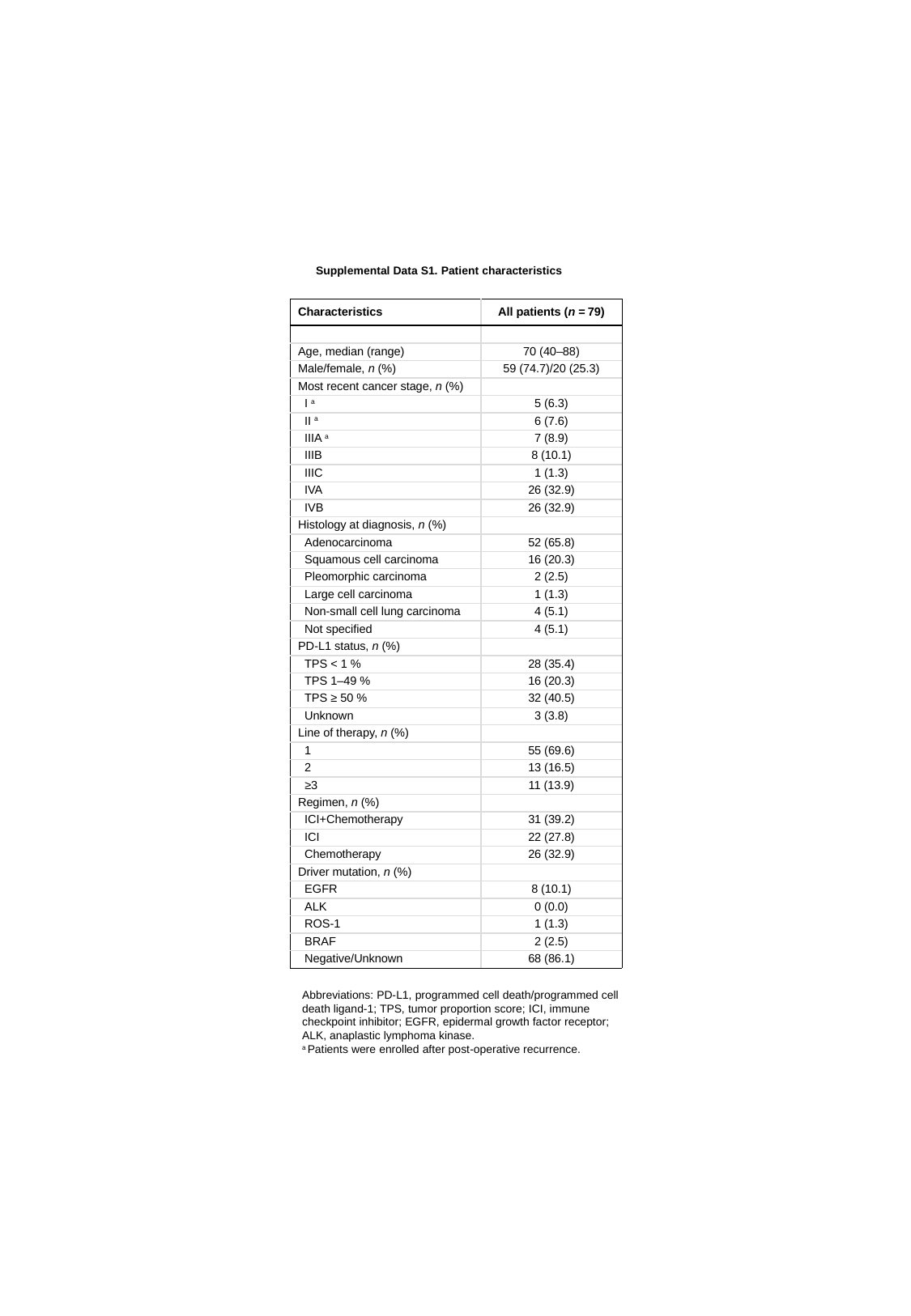

Supplemental Data S1. Patient characteristics
| Characteristics | All patients (n = 79) |
| --- | --- |
| | |
| Age, median (range) | 70 (40–88) |
| Male/female, n (%) | 59 (74.7)/20 (25.3) |
| Most recent cancer stage, n (%) | |
| I a | 5 (6.3) |
| II a | 6 (7.6) |
| IIIA a | 7 (8.9) |
| IIIB | 8 (10.1) |
| IIIC | 1 (1.3) |
| IVA | 26 (32.9) |
| IVB | 26 (32.9) |
| Histology at diagnosis, n (%) | |
| Adenocarcinoma | 52 (65.8) |
| Squamous cell carcinoma | 16 (20.3) |
| Pleomorphic carcinoma | 2 (2.5) |
| Large cell carcinoma | 1 (1.3) |
| Non-small cell lung carcinoma | 4 (5.1) |
| Not specified | 4 (5.1) |
| PD-L1 status, n (%) | |
| TPS < 1 % | 28 (35.4) |
| TPS 1–49 % | 16 (20.3) |
| TPS ≥ 50 % | 32 (40.5) |
| Unknown | 3 (3.8) |
| Line of therapy, n (%) | |
| 1 | 55 (69.6) |
| 2 | 13 (16.5) |
| ≥3 | 11 (13.9) |
| Regimen, n (%) | |
| ICI+Chemotherapy | 31 (39.2) |
| ICI | 22 (27.8) |
| Chemotherapy | 26 (32.9) |
| Driver mutation, n (%) | |
| EGFR | 8 (10.1) |
| ALK | 0 (0.0) |
| ROS-1 | 1 (1.3) |
| BRAF | 2 (2.5) |
| Negative/Unknown | 68 (86.1) |
Abbreviations: PD-L1, programmed cell death/programmed cell death ligand-1; TPS, tumor proportion score; ICI, immune checkpoint inhibitor; EGFR, epidermal growth factor receptor; ALK, anaplastic lymphoma kinase.
a Patients were enrolled after post-operative recurrence.

## Slide 2
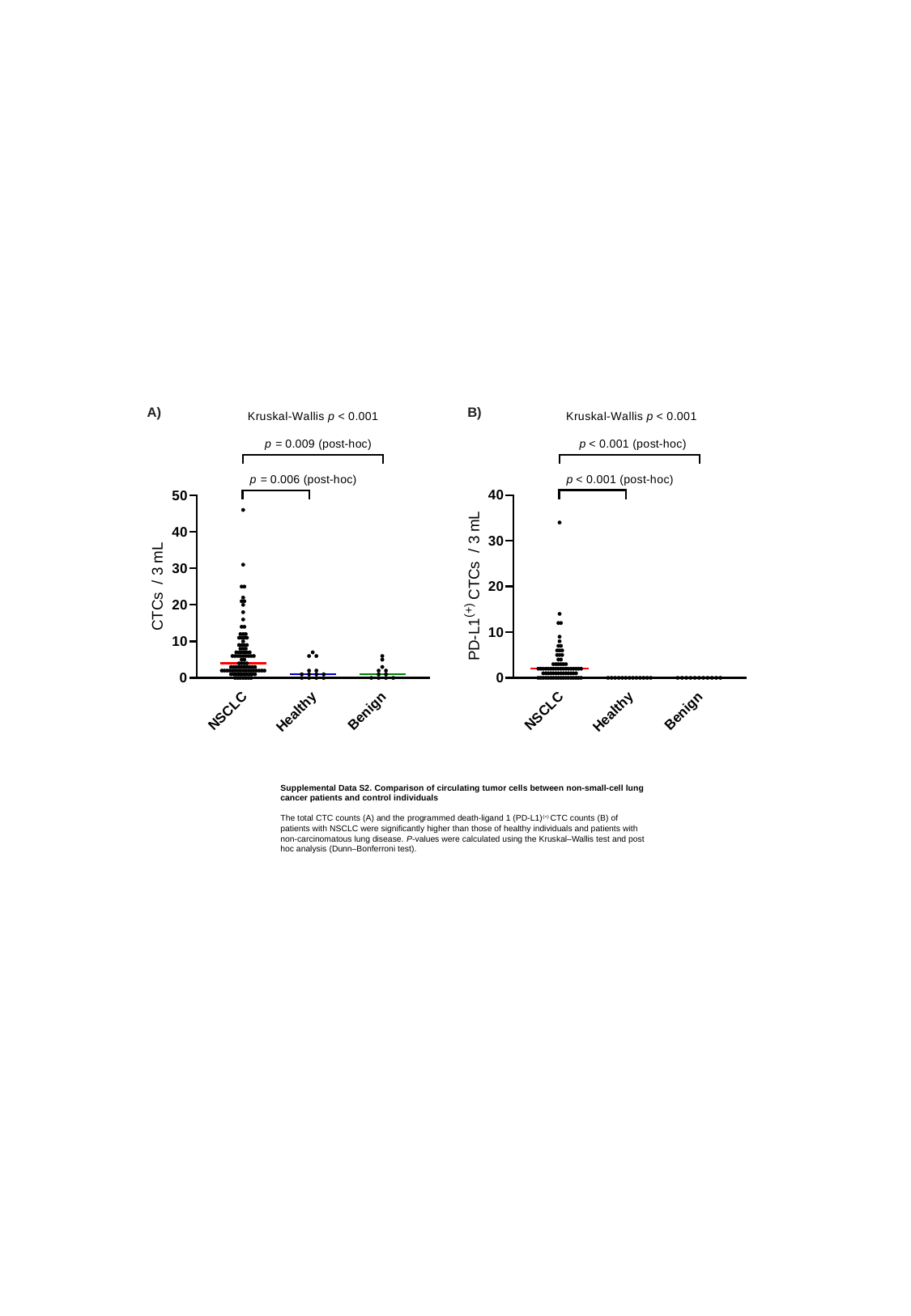

A)
B)
Supplemental Data S2. Comparison of circulating tumor cells between non-small-cell lung cancer patients and control individuals
The total CTC counts (A) and the programmed death-ligand 1 (PD-L1)(+) CTC counts (B) of patients with NSCLC were significantly higher than those of healthy individuals and patients with non-carcinomatous lung disease. P-values were calculated using the Kruskal–Wallis test and post hoc analysis (Dunn–Bonferroni test).

## Slide 3
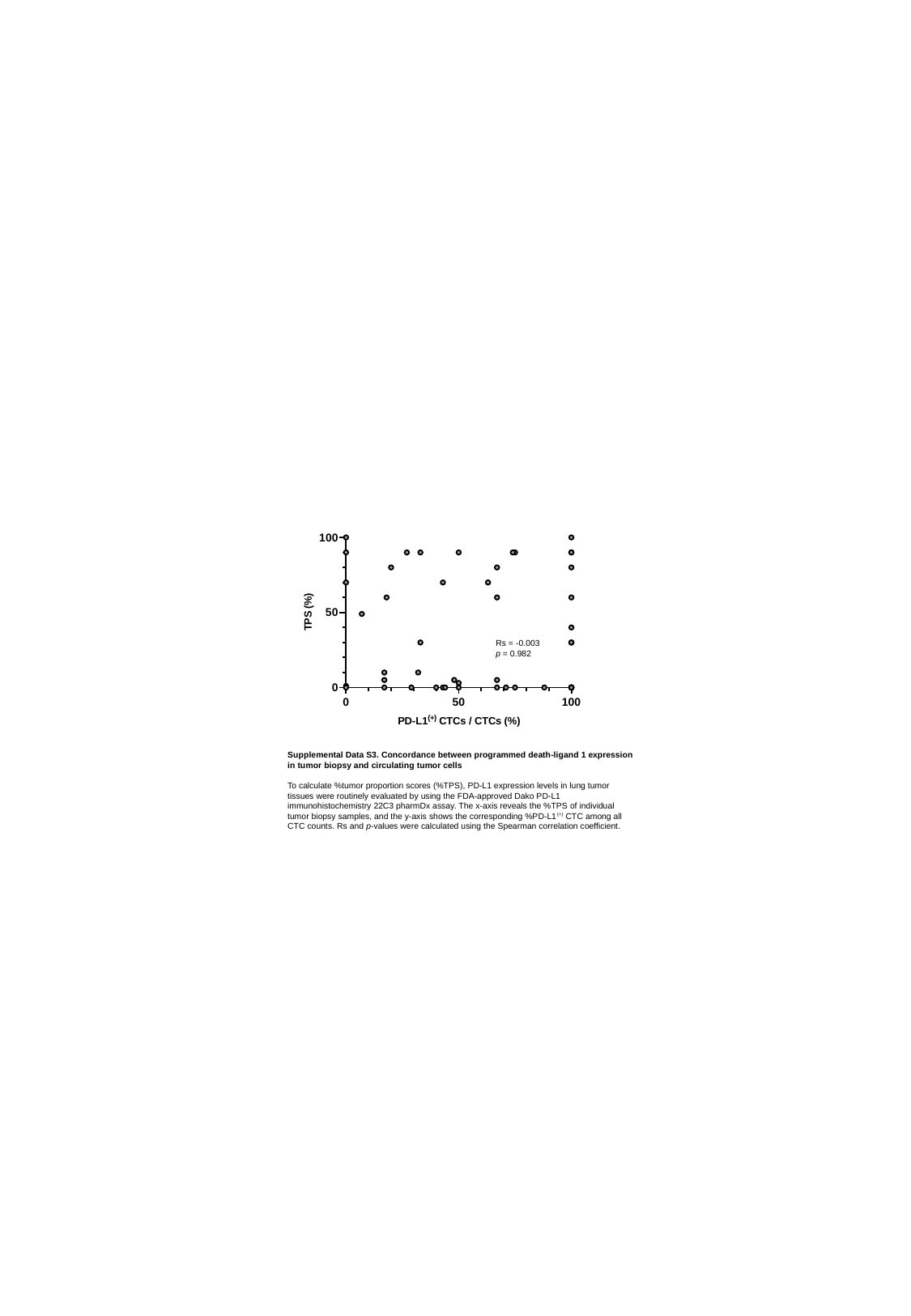

Rs = -0.003
 p = 0.982
Supplemental Data S3. Concordance between programmed death-ligand 1 expression in tumor biopsy and circulating tumor cells
To calculate %tumor proportion scores (%TPS), PD-L1 expression levels in lung tumor tissues were routinely evaluated by using the FDA-approved Dako PD-L1 immunohistochemistry 22C3 pharmDx assay. The x-axis reveals the %TPS of individual tumor biopsy samples, and the y-axis shows the corresponding %PD-L1(+) CTC among all CTC counts. Rs and p-values were calculated using the Spearman correlation coefficient.

## Slide 4
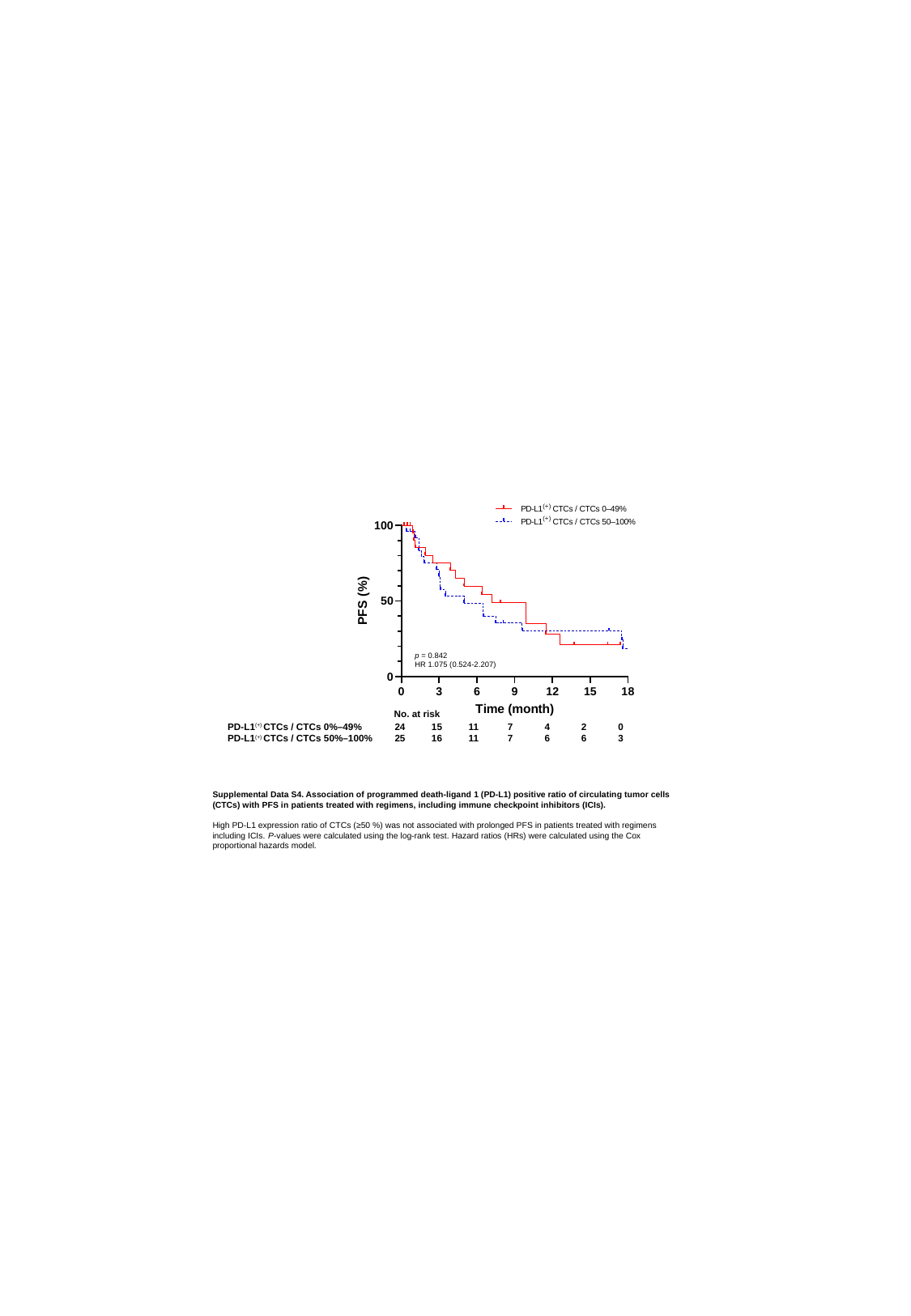

p = 0.842
HR 1.075 (0.524-2.207)
No. at risk
PD-L1(+) CTCs / CTCs 0%–49%
PD-L1(+) CTCs / CTCs 50%–100%
24 15 11 7 4 2 0
25 16 11 7 6 6 3
Supplemental Data S4. Association of programmed death-ligand 1 (PD-L1) positive ratio of circulating tumor cells (CTCs) with PFS in patients treated with regimens, including immune checkpoint inhibitors (ICIs).
High PD-L1 expression ratio of CTCs (≥50 %) was not associated with prolonged PFS in patients treated with regimens including ICIs. P-values were calculated using the log-rank test. Hazard ratios (HRs) were calculated using the Cox proportional hazards model.

## Slide 5
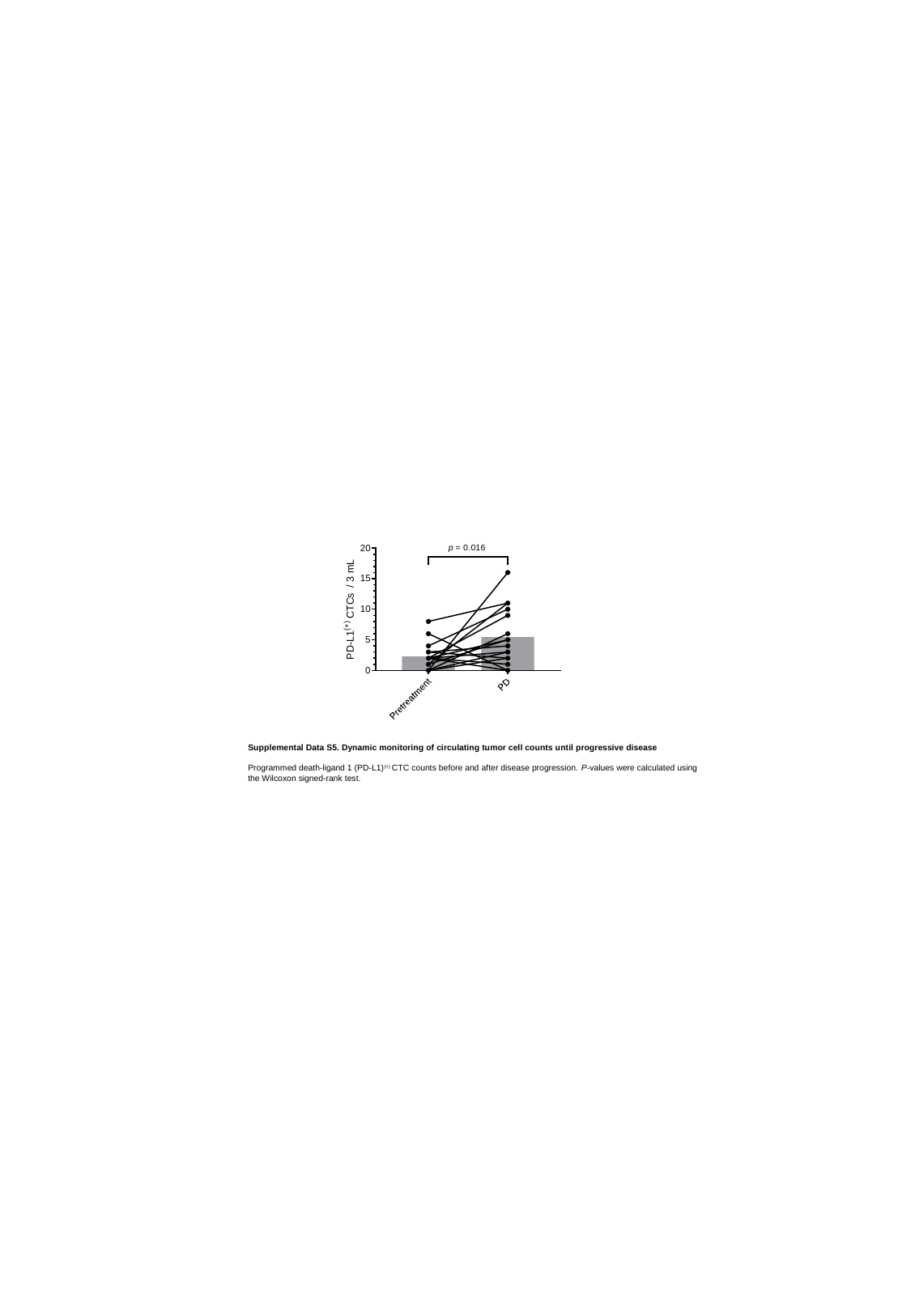

Supplemental Data S5. Dynamic monitoring of circulating tumor cell counts until progressive disease
Programmed death-ligand 1 (PD-L1)(+) CTC counts before and after disease progression. P-values were calculated using the Wilcoxon signed-rank test.

## Slide 6
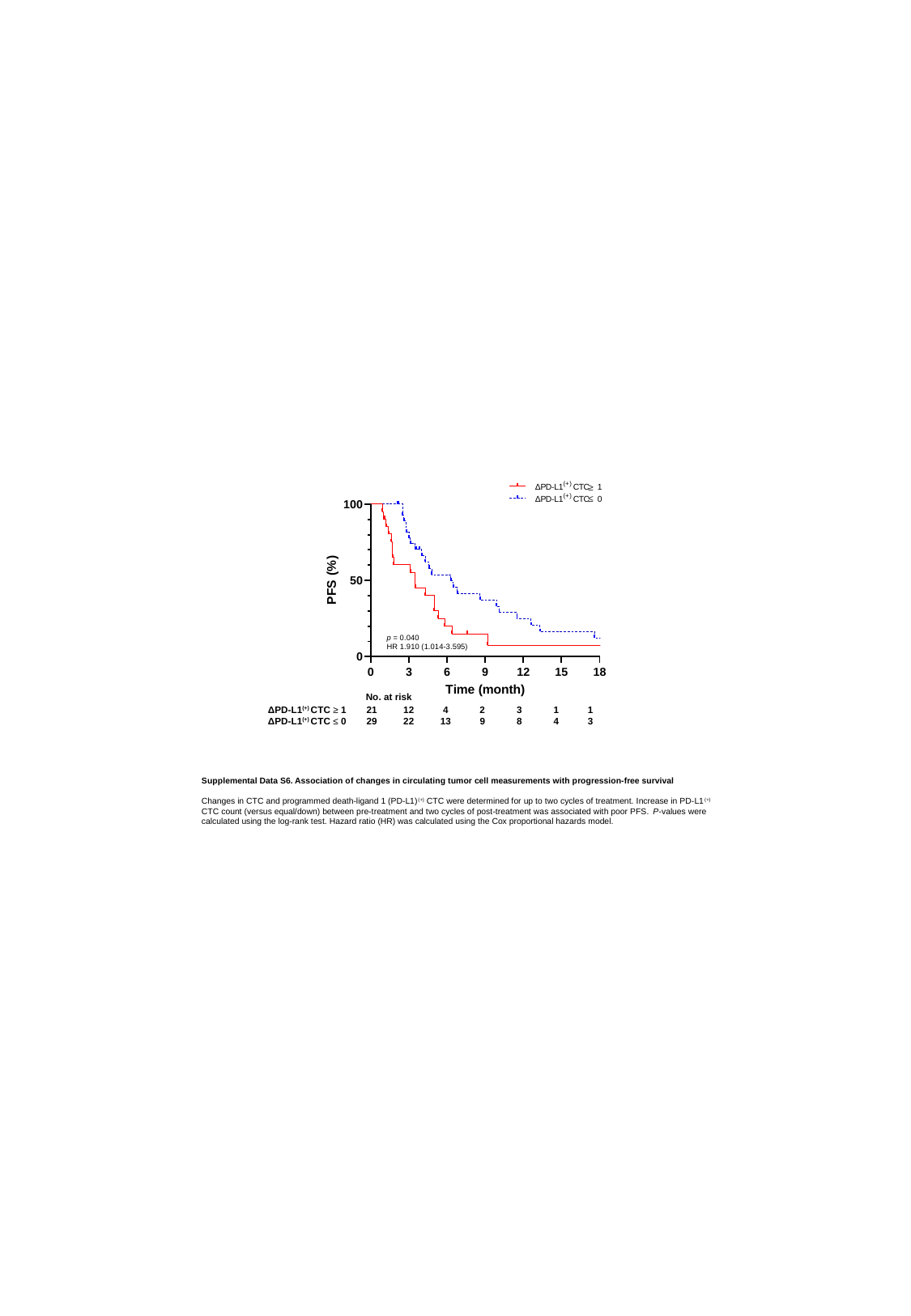

≥
≤
p = 0.040
HR 1.910 (1.014-3.595)
No. at risk
ΔPD-L1(+) CTC ≥ 1
ΔPD-L1(+) CTC ≤ 0
21 12 4 2 3 1 1
29 22 13 9 8 4 3
Supplemental Data S6. Association of changes in circulating tumor cell measurements with progression-free survival
Changes in CTC and programmed death-ligand 1 (PD-L1)(+) CTC were determined for up to two cycles of treatment. Increase in PD-L1(+) CTC count (versus equal/down) between pre-treatment and two cycles of post-treatment was associated with poor PFS. P-values were calculated using the log-rank test. Hazard ratio (HR) was calculated using the Cox proportional hazards model.
